# Supplementary figures and images for: Saccharibacteria (TM7), but not other bacterial taxa, are associated with childhood caries regardless of age in a South China population
Source: PeerJ. 2023 Jun 26;11:e15605. doi: 10.7717/peerj.15605 (PMC10309052; doi:10.7717/peerj.15605)

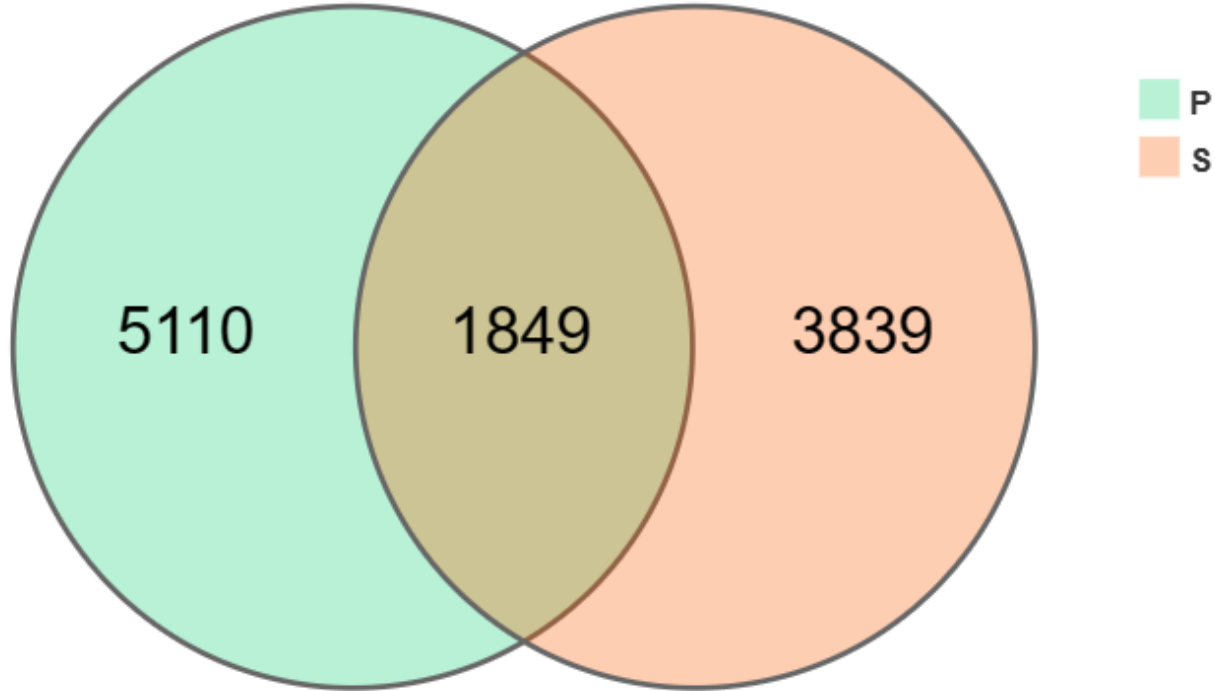

Supplement: Supplemental Information 1 [file peerj-11-15605-s001.pdf]

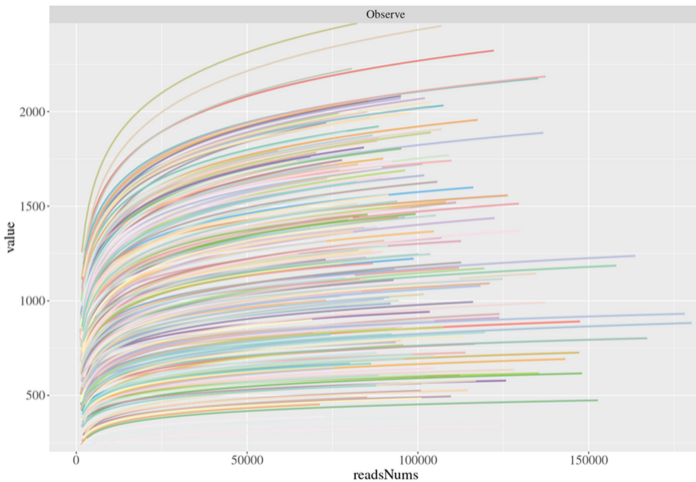

A

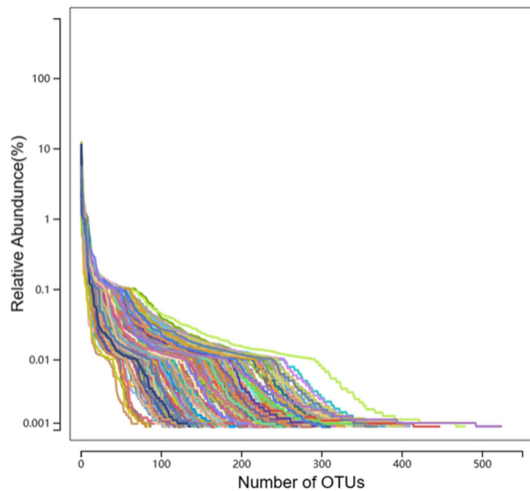

B

Supplement: Supplemental Information 2 [file peerj-11-15605-s002.pdf]

A

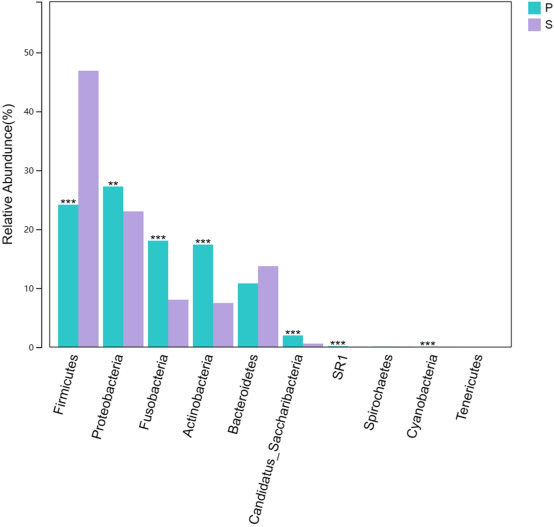

B

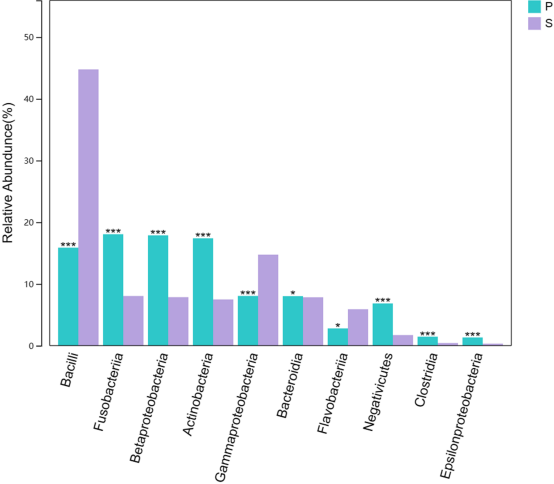

C

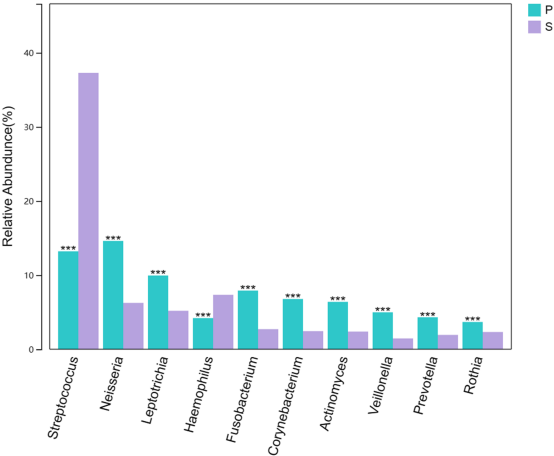

Supplement: Supplemental Information 3 — (A) phyla, (B) class, and (C) genera among plaque (P) and saliva (S) groups. [file peerj-11-15605-s003.pdf]

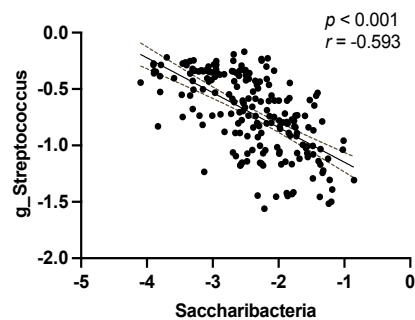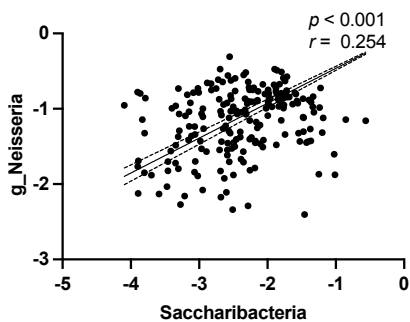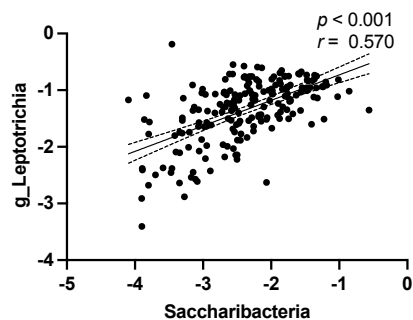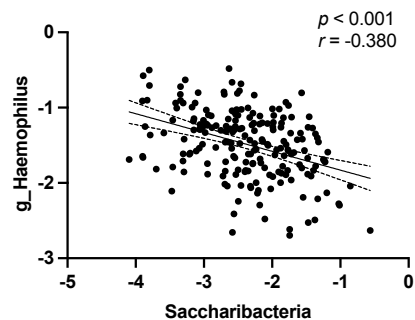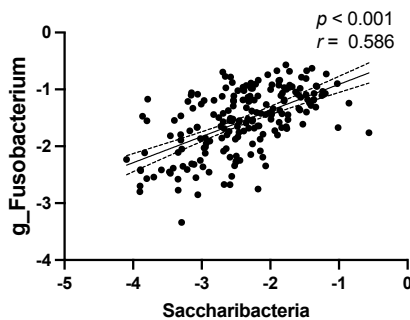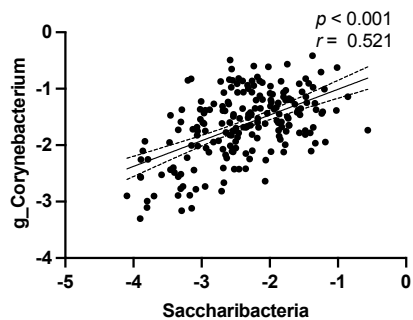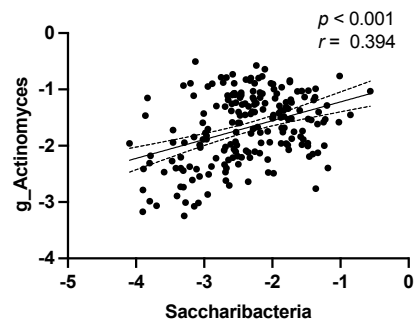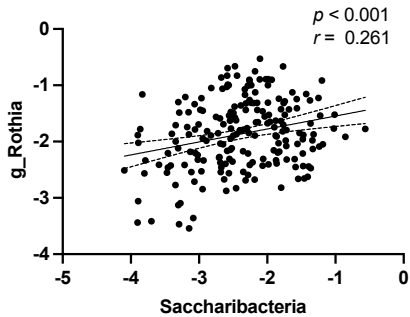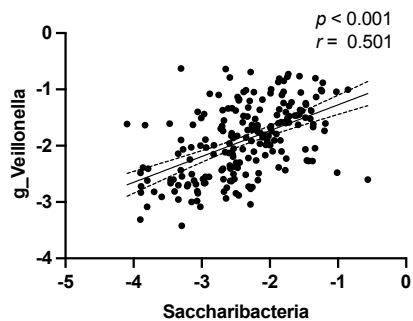

Supplement: Supplemental Information 4 [file peerj-11-15605-s004.pdf]
