# Supplementary material for: Saccharibacteria (TM7), but not other bacterial taxa, are associated with childhood caries regardless of age in a South China population
Source: PeerJ. 2023 Jun 26;11:e15605. doi: 10.7717/peerj.15605 (PMC10309052; doi:10.7717/peerj.15605)
Supplement: Supplemental Information 5 [file peerj-11-15605-s005.docx]

**Table S1** Plaque bacteria correlated with dt in the female and male groups.

| **Group** | **feature** | **PF** | | |  | **PM** | | |
| --- | --- | --- | --- | --- | --- | --- | --- | --- |
|  |  | *R^2^* | *p* | *q* |  | *R^2^* | *p* | *q* |
| **PF** | *g__Moryella* | 0.009 | 0.017 | 0.213 |  | - | - | - |
|  | *g__Veillonella* | 0.045 | 0.013 | 0.213 |  | - | - | - |
|  | *f__Veillonellaceae* | 0.050 | 0.007 | 0.100 |  | - | - | - |
|  | *o__Clostridiales* | 0.060 | 0.001 | 0.016 |  | - | - | - |
|  | *o__Lactobacillales* | -0.040 | 0.037 | 0.234 |  | - | - | - |
|  | *o__Pseudomonadales* | -0.010 | 0.049 | 0.234 |  | - | - | - |
|  | *c__Clostridia* | 0.060 | 0.001 | 0.010 |  | - | - | - |
|  | *c__TM7.3* | 0.036 | 0.002 | 0.011 |  | - | - | - |
|  | *c__Bacilli* | -0.040 | 0.049 | 0.194 |  | - | - | - |
|  | *p__TM7* | 0.036 | 0.002 | 0.015 |  | - | - | - |
| **PM** | *g__Porphyromonas* | - | - | - |  | -0.018 | 0.003 | 0.095 |
|  | *g__Fusobacterium* | - | - | - |  | -0.042 | 0.004 | 0.095 |
|  | *g__Neisseria* | - | - | - |  | -0.043 | 0.009 | 0.116 |
|  | *g__Acinetobacter* | - | - | - |  | -0.008 | 0.008 | 0.116 |
|  | *g__Granulicatella_f__Carnobacteriaceae* | - | - | - |  | -0.012 | 0.021 | 0.214 |
|  | *f__Fusobacteriaceae* | - | - | - |  | -0.042 | 0.004 | 0.129 |
|  | *f__Porphyromonadaceae* | - | - | - |  | -0.017 | 0.013 | 0.152 |
|  | *f__Neisseriaceae* | - | - | - |  | -0.045 | 0.009 | 0.152 |
|  | *f__Carnobacteriaceae* | - | - | - |  | -0.012 | 0.021 | 0.188 |
|  | *o__Neisseriales* | - | - | - |  | -0.045 | 0.009 | 0.190 |
|  | *c__Betaproteobacteria* | - | - | - |  | -0.044 | 0.009 | 0.122 |
|  | *c__Actinobacteria* | - | - | - |  | 0.045 | 0.049 | 0.194 |
|  | *p__Actinobacteria* | - | - | - |  | 0.045 | 0.049 | 0.125 |

Abbreviations: PF, Females' supragingival plaque; PM, Males' supragingival plaque.
