# Supplementary material for: Saccharibacteria (TM7), but not other bacterial taxa, are associated with childhood caries regardless of age in a South China population
Source: PeerJ. 2023 Jun 26;11:e15605. doi: 10.7717/peerj.15605 (PMC10309052; doi:10.7717/peerj.15605)
Supplement: Supplemental Information 6 [file peerj-11-15605-s006.docx]

**Table S2** The saliva bacteria correlated with dt in male group.

| **Group** | **feature** | ***R^2^*** | ***p*** | ***q*** |
| --- | --- | --- | --- | --- |
| **SM** | *g__Actinomyces* | 0.038 | 0.003 | 0.077 |
|  | *g__Corynebacterium* | 0.031 | 0.020 | 0.208 |
|  | *g__Rothia* | 0.028 | 0.022 | 0.208 |
|  | *g__Granulicatella_f__Carnobacteriaceae* | -0.027 | 0.025 | 0.208 |
|  | *g__Selenomonas* | 0.007 | 0.034 | 0.218 |
|  | *f__Actinomycetaceae* | 0.042 | 0.003 | 0.054 |
|  | *f__Corynebacteriaceae* | 0.031 | 0.020 | 0.147 |
|  | *f__Micrococcaceae* | 0.028 | 0.022 | 0.147 |
|  | *f__.Weeksellaceae.* | -0.048 | 0.029 | 0.147 |
|  | *f__Carnobacteriaceae* | -0.027 | 0.025 | 0.147 |
|  | *f__Comamonadaceae* | -0.007 | 0.020 | 0.147 |
|  | *f__Veillonellaceae* | 0.021 | 0.039 | 0.174 |
|  | *o__Actinomycetales* | 0.064 | 0.003 | 0.032 |
|  | *o__Clostridiales* | 0.022 | 0.026 | 0.185 |
|  | *c__TM7.3* | 0.022 | 0.001 | 0.019 |
|  | *c__Actinobacteria* | 0.064 | 0.003 | 0.021 |
|  | *c__Clostridia* | 0.022 | 0.026 | 0.123 |
|  | *p__TM7* | 0.022 | 0.001 | 0.013 |
|  | *p__Actinobacteria* | 0.064 | 0.003 | 0.014 |

Abbreviations: SM, Males' unstimulated saliva.
